# A Morphospace for Reef Fishes: Elongation Is the Dominant Axis of Body Shape Evolution

**DOI:** 10.1371/journal.pone.0112732

**Published:** 2014-11-19

**Authors:** Thomas Claverie, Peter C. Wainwright

**Affiliations:** 1 CUFR de Mayotte, Route nationale 3, 97660 Dembeni, France; 2 Department of Evolution and Ecology, University of California Davis, Davis, California, United States of America; Ecole Normale Supérieure de Lyon, France

## Abstract

Tropical reef fishes are widely regarded as being perhaps the most morphologically diverse vertebrate assemblage on earth, yet much remains to be discovered about the scope and patterns of this diversity. We created a morphospace of 2,939 species spanning 56 families of tropical Indo-Pacific reef fishes and established the primary axes of body shape variation, the phylogenetic consistency of these patterns, and whether dominant patterns of shape change can be accomplished by diverse underlying changes. Principal component analysis showed a major axis of shape variation that contrasts deep-bodied species with slender, elongate forms. Furthermore, using custom methods to compare the elongation vector (axis that maximizes elongation deformation) and the main vector of shape variation (first principal component) for each family in the morphospace, we showed that two thirds of the families diversify along an axis of body elongation. Finally, a comparative analysis using a principal coordinate analysis based on the angles among first principal component vectors of each family shape showed that families accomplish changes in elongation with a wide range of underlying modifications. Some groups such as Pomacentridae and Lethrinidae undergo decreases in body depth with proportional increases in all body regions, while other families show disproportionate changes in the length of the head (e.g., Labridae), the trunk or caudal region in all combinations (e.g., Pempheridae and Pinguipedidae). In conclusion, we found that evolutionary changes in body shape along an axis of elongation dominates diversification in reef fishes. Changes in shape on this axis are thought to have immediate implications for swimming performance, defense from gape limited predators, suction feeding performance and access to some highly specialized habitats. The morphological modifications that underlie changes in elongation are highly diverse, suggesting a role for a range of developmental processes and functional consequences.

## Introduction

Repeated patterns of trait variation across species, such as wing analogies, can be of major interest for evolutionary biologists since they may reflect shared underlying evolutionary processes, such as flight mechanical constraints. One such repeated pattern appears to be body elongation in vertebrates [1]–[5]. Elongation has been shown to be the dominant axis of shape diversification in some clades [2], but few studies specifically address the repetition of this pattern in vertebrates. Here, we propose to investigate the prevalence of elongation in teleost fishes. Teleosts are a species-rich clade of vertebrates with more than 32 000 species among little more than 60 000 vertebrate species [6]. As such, this major group is of prime interest to study occurrences and processes of elongation in vertebrates.

**Figure 1:**
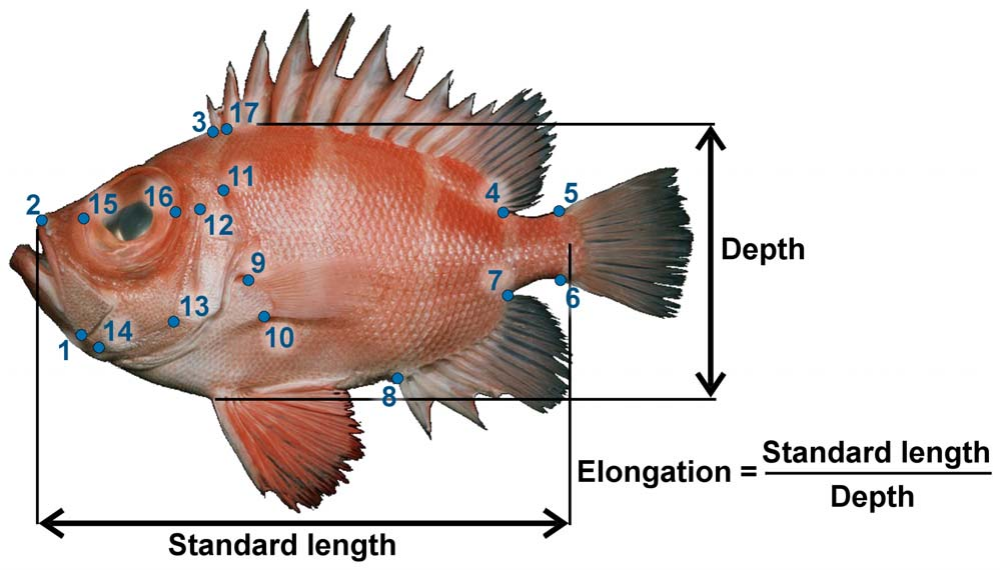
Landmarks that were digitized to characterize body shape of Indo-Pacific shore fishes. There were 17 landmarks. Landmarks 1, 2 and 11–17 represent important landmark related to feeding mechanics while landmarks 3–10 represent important landmarks for locomotion mechanics. Maximum body depth and standard length were also measured to calculate body elongation index.

Previous phylogenetically broad investigations suggest that elongation (measured as the ratio of body length by body depth, Fig. 1) is a major morphological trend across teleost species [3]–[5]. Studies on overall body shape or shape of part of the body (often part of the cranium), have found that elongation is the principal axis of shape diversity within clades of Pomacentridae [7]–[10], Balistidae [11], Cichlidae [12], Anguilliformes [13], [14] and Labridae [15]. A major role for elongation has even been described several times intraspecifically (e.g. in Cichlidae [16]). However, for some of these studies, the number of species representing larger clades includes only a small fraction of the group (often less than 1% of the recognized species), and only a handful of groups have been investigated. Therefore, it remains unclear whether elongation is a major and repeated pattern of body shape diversification in teleosts.

In this study, we addressed two questions. First, is elongation a major trend in shape diversification in reef fishes? Second, do we see a common underlying anatomy of changes in elongation or is there diversity in this anatomy that leads to a convergent trend in fish shape diversification? We investigate these questions using a tropical reef fish morphospace based on lateral-view photographs (Fig. 1) of 2939 Indo-Pacific species belonging to 56 families, or roughly 10% of all teleosts. Tropical marine fishes that live in association with reefs are widely regarded as being among the most morphologically diverse assemblages of fishes on earth and can, as such, be used to represent variation in teleosts. Based on previous work on fish morphology, for the first question, we can hypothesize that elongation is a major repeated axis of shape diversification in teleosts. To address the second question, we explore whether there is evidence for divergence among groups in how the fish body becomes elongate, or deep-bodied, during evolution. Indeed, although variation in elongation appears to occur regularly among vertebrates, the underlying anatomical basis of elongation can differ greatly. For example, in mammals and birds, the head appears to be the dominant body region for variation in elongation, while in Actinopterygii (ray-finned fishes) and in amphibians there is more variation in the post cranial region [1]. Furthermore, in bony fishes, elongation appears dominant in the precaudal region in Sarcopterygii but in the caudal region in chondrichthyans and actinopterygians [17]. However, a previous survey of actinopterygians suggested that abdominal vs caudal elongation could be clade specific [5].

Finally, recent advances in the phylogenetics of teleosts and particularly acanthomorphs make it clear that far from being a single monophyletic group, reef fishes are highly polyphyletic [18]–[20]. The vast majority of reef fish lineages are acanthomorphs (spiny-rayed fishes), but the modern reef fish fauna reflects the accumulation of many lineages that have independently made the transition to reef habitats over the past 100 million years [15], [20]–[22]. Unfortunately, no comprehensive species-level phylogeny exists for modern reef fishes yet, so it is not possible to match our sample of reef fishes with a phylogenetic hypothesis that would allow the exploration of a number of additional questions about the evolutionary history of body shape.

## Methods

### Specimen sampling and family grouping

A total of 2939 species from 56 families of Indo-Pacific fishes were included in this study (Table 1). Most of these were acanthomorphs, with the exception of some clupeids, engraulids, and synodontids. Anguiliform species such as the Muraenidae were not included in this study because all specimens were photographed in a bent posture and therefore were inapproriate for morphometric analysis of body shape. Our sample represents nearly 10% of described teleost species and over 17% of acanthomorph species. Photos were obtained from the online picture repository of photographs taken by Dr. Jack Randall at the Bishop Museum (http://pbs.bishopmuseum.org/images/JER/images.asp). The numbers of species included in that study was therefore constrained by their availability in that picture repository. Analyses were conducted with the lateral view photograph of a single adult individual of each species.

**Table 1:** Families included in the present study with numbers of species sampled and the number of species recognized in each family by the catalog of fishes [6].

Family ID	Family name	Number of species sampled	Number of valid species	Family ID	Family name	Number of species sampled	Number of valid species
1	Acanthuridae	59	85	29	Lutjanidae	77	133
2	Antennariidae	13	48	30	Malacanthidae	11	46
3	Apogonidae	138	347	31	Monacanthidae	35	109
4	Atherinidae+	11	71	32	Mugilidae	18	72
5	Balistidae	25	41	33	Mullidae	38	83
6	Batrachoididae^#^	4	83	34	Nemipteridae	39	69
7	Belonidae++	9	38	35	Opistognathidae+	14	81
8	Blenniidae	167	404	36	Ostraciidae	25	27
9	Bythitidae+	15	209	37	Pempheridae	23	30
10	Callionymidae	49	189	38	Pinguipedidae	27	84
11	Carangidae	52	148	39	Platycephalidae	24	81
12	Chaetodontidae	97	131	40	Plesiopidae	15	49
13	Cheilodactylidae	10	27	41	Pomacanthidae	62	88
14	Cirrhitidae	20	35	42	Pomacentridae	216	390
15	Clupeidae+	30	195	43	Priacanthidae	11	19
16	Diodontidae++	9	18	44	Pseudochromidae	50	152
17	Engraulidae+	12	146	45	Sciaenidae	17	291
18	Ephippidae++	4	15	46	Scombridae	18	53
19	Epinephelinae	115	233	47	Scorpaenidae+	61	351
20	Gobiesocidae+	10	162	48	Serranidae	76	300
21	Gobiidae	547	1772	49	Siganidae+	21	32
22	Haemulidae	29	132	50	Sparidae+	21	134
23	Hemiramphidae	24	61	51	Sphyraenidae	12	29
24	Holocentridae	52	84	52	Synanceiidae+	20	75
25	Kyphosidae^#^	8	51	53	Syngnathidae	18	338
26	Labridae	334	614	54	Synodontidae	22	69
27	Leiognathidae	17	50	55	Tetraodontidae	56	190
28	Lethrinidae	28	41	56	Tripterygiidae+	24	168

+ represents the poorly sampled taxa (<20% of total accepted number of species),

++ represent taxa with less than 10 species sampled and ^#^ represent families poorly sampled and with less than 10 species.

Species were grouped into monophyletic families using the Catalog of Fishes [6], with some modifications to reflect recent phylogenetic discoveries. Departures from the Catolog of Fishes concerned Serranidae, Caesionidae, Tetrarogidae, Sebastidae, Scaridae and Microdesmidae. Serranidae was divided in two groups: Serranidae (including Serraninae and Anthiinae, mainly the latter in this work) and Ephinephelidae [23]. Caesionidae appears to be nested within Lutjanidae and was included in that family [24]. Tetrarogidae was included in Synanceiidae [25], Sebastidae was included with Scorpaenidae [25], Scaridae was included with Labridae [26], [27], and Microdesmidae was included in Gobiidae [28], [29].

### Morphometrics

Landmark-based geometric morphometric methods [30] were used to quantify body shape from 17 landmarks (Fig. 1, Table 2). Landmarks were positioned to capture major body regions and functionally significant features relevant to feeding and locomotion. Landmarks were limited to those representing operationally homologous features that could be found in every species. Landmark positions are described in Table 2 (Fig. 1). For one family, the Syngnathidae, the anal fins were either not present or vestigial and situated next to the anus on the anterior side of the egg pouch in some tail-brooder (e.g. *Syngnathus leptorhynchus*). In syngnathids, landmarks 7 and 8 were situated side by side next to the anus and on the anterior edge of the egg pouch in tail-brooders or on the posterior edge of the pouch in abdominal-brooders. The coordinates of these 17 landmarks were digitized using TPSdig [31].

**Table 2:** Disposition of landmarks on specimens.

Landmarks number	Description of its position
1	Posterior-ventral margin of the distal arm of the maxilla
2	Most anterior proximal limit between the premaxilla and the head of the maxilla
3	Insertion of the most anterior ray of the dorsal fin on the body
4	Insertion of the most posterior ray of the dorsal fin on the body
5	Dorsal insertion of the caudal fin
6	Ventral insertion of the caudal fin
7	Insertion of the first ray of the anal fin
8	Insertion of the last ray of the anal fin
9	Anterior-dorsal insertion of the pectoral fin
10	Posterior-ventral insertion of the pectoral fin
11	Joint between the post-temporal and supracleithrum. Movement at this joint accompanies cranial elevation during prey capture. We located this landmark at the intersection between the lateral line and the posterior-dorsal edge of the operculum
12	Most dorsal point on the preopercle
13	Inflection in the deep preopercular bend
14	Anterior tip of the preopercle
15	Most anterior point on the eye
16	Most posterior point on the eye
17	In association with landmark 11, was used as an estimate of epaxial muscle height and was situated on the dorsal edge of the fish vertically above landmark 11

Landmark configurations of every fish image were digitally superimposed and morphological information was extracted. Superimposition consists of rotating, translating and rescaling the landmark configurations to remove variation due to relative fish size and position on pictures. This procedure requires the construction of a morphospace, which represents a multivariate space defined by the type of forms studied and the number of landmark coordinates, and is used to quantify differences among specimens [32]. This procedure was done using the generalized Procrustes superimposition method [33]. Tangential Procrustes residuals (i.e. superimposed coordinates minus consensus coordinates) were used as the shape data for subsequent analyses [34]. The R packages Geomorph and Morpho were used for these procedures [35].

Geometric morphometric methods assume a limited spread of specimens in the morphospace, but we investigated extensive morphological variation such that a potential breach of this assumption had to be investigated. Most statistics in geometric morphometrics are based on Euclidean space while shape data belongs to what is called the “Kendal shape space” [32]. Therefore a projection of data from the Kendal shape space to the Euclidean space is required before analyses can be done. However, curvature of both spaces are not the same and during projection distortion of specimen position in the morphospace might occur in a manner similar to the way that landscapes get distorted when viewed through a fisheye lens. The error made by such projection was estimated by comparing pairwise Procrustes distances (i.e. shape difference) among landmark configurations from both spaces (Kendall and Euclidean) using a correlation [36]. This was done using TPSsmall [37]. The Pearson's product moment correlation between distances measured in the two spaces was very high (p<0.001, r = 1.00). Thus, standard statistics could be applied to this dataset.

### Body shape variation among species

We summarized trends in fish shape using a principal component analysis (PCA) on the shape variables (Procrustes residuals) of all species. Thin-plate spline interpolation functions were used to compute deformation grids and represent shape variation across species [34]. A broken stick procedure was used as a rule of thumb to decide how many principal components (PC) were relevant and significant for interpretation [38]. Investigation of shape variation described by the significant PCs was used to investigate body shape variation across the entire sample and within each of the 56 families.

Morphological diversification probably does not occur in the same way in every family, but it is possible that there are common tendencies or repeating patterns. We compared the primary axes of diversification across families using a combination of ordination methods. The first principal component of shape in each family was used to represent the trend in morphological diversification. The first PC of shape for each family is a vector passing through the cloud of representative species in the direction of their maximum spread in the morphospace. We therefore performed separate Procrustes superimpositions and PCA's for each family and extracted the coordinates of each PC1. We then calculated the pairwise angles among these vectors to characterize the differences in diversification among families. These angles took values between 0 degrees (parallel) and 90 degrees (orthogonal). Finally, we performed a principal coordinate analysis (PCoA [39]) on the pairwise angles dissimilarity matrix to identify which families had similar axes of variation (e.g. parallel diversification) and whether clustering of families was present. These analyses were performed using custom functions written in R [35]. These functions are provided as File S1.

### Elongation and maximum axis of shape variation

Several taxonomically focused studies of fish shape found deformation associated with body elongation was a major axis of shape variation [3], [4], [7], [9], [11], [12]. To assess the recurrence of body elongation as the dominant axis of shape change in different families from our sample, we determined how strongly elongation is related to the main axis of variation (PC1) in each family and how much of the shape variation is explained by elongation within each family.

For each family, we measured the angle between two vectors in the morphospace: the vector describing maximum shape variation among species, PC1, and the vector indicating the direction of body elongation among species. We defined ‘elongation’ as the ratio between fish standard length and maximum body depth (Fig. 1). The elongation vector in the morphospace of each family was the vector that maximized differences in elongation among species. The coordinates of this vector were calculated with a modified redundancy analysis (RDA) that combined regression and PCA. Conceptually, a RDA is a multivariate multiple linear regression followed by a PCA on the matrix of regression fitted values [39], [40]. The method is used to find the maximum axis of variance in a multivariate dataset along a gradient also defined by a multivariate dataset. We wanted to find the vector that describes maximum elongation variance in the morphospace. Therefore, we used a univariate multiple linear regression to calculate shape-fitted values using elongation as the independent variable. We then performed a PCA on these fitted values to extract the coordinates of the first eigenvector that describes the axis of maximum elongation in the morphospace of each family. We subsequently calculated the angle between the axis of maximum elongation and PC1 for each family. Significance was assessed using a permutation procedure.

The permutation procedure tested the null hypothesis that elongation and PC1 for each family were not correlated. We randomly shuffled elongation variables for each specimen in a family (random sampling without replacement) and calculated the new coordinates of the elongation vector using the procedure described above. We then calculated the angle between PC1 and this new elongation vector. We performed this randomization 1000 times to construct a distribution of angles. Based on that distribution and the angle between PC1 and elongation we could estimate the probability of independence between elongation and PC1 for each family. R codes used for this procedure are given in File S1.

## Results

### Elongation is the major axis of shape variation

**Figure 2:**
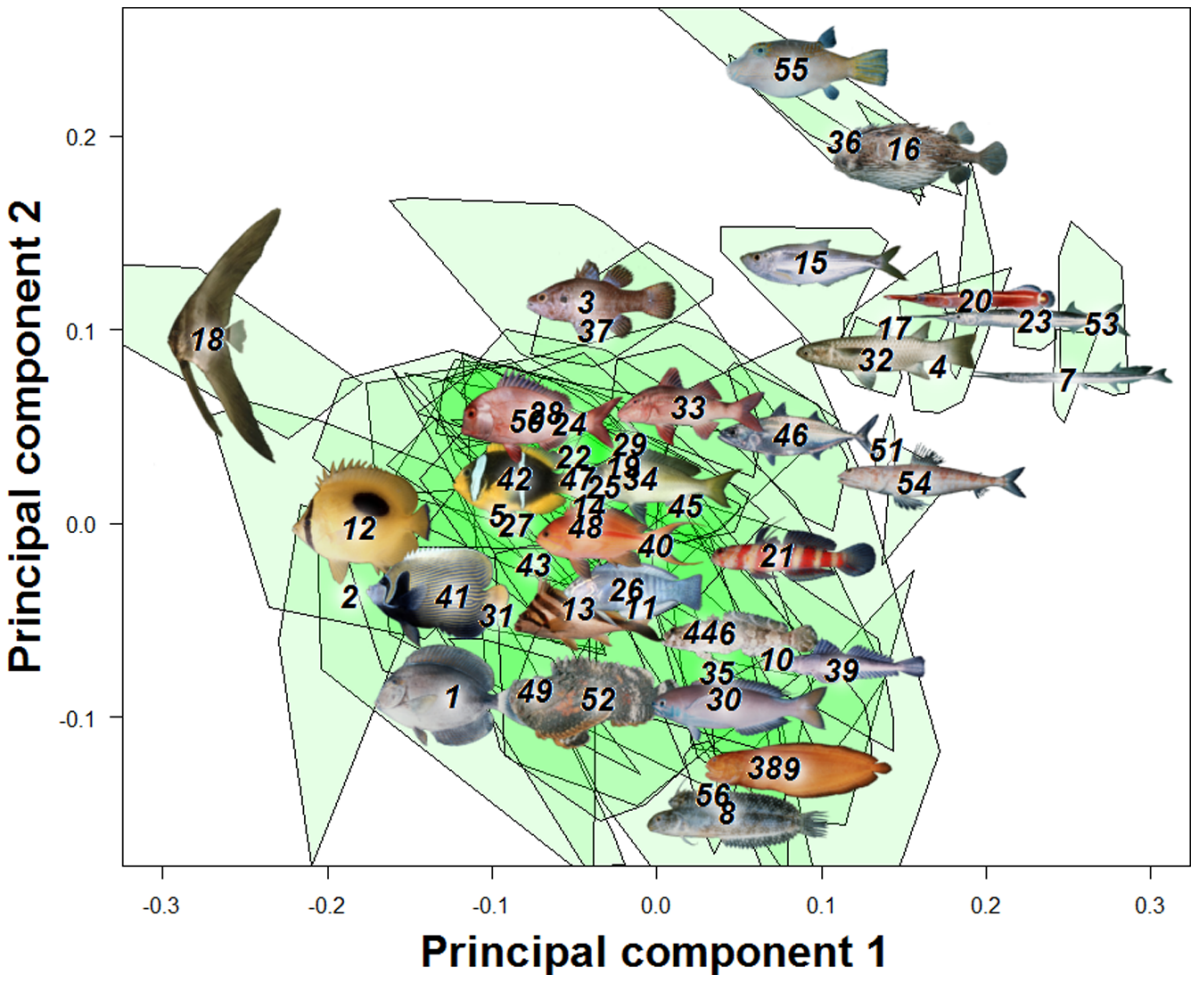
A principal component analysis (PCA) on overall fish shape data showed four main axes of shape deformation representing respectively 32.1%, 26.22%, 13.23% and 6.87% of the overall shape variation in reef fishes (A & B). Fish pictures and family numbers are placed on the centroid of each family and used to identify their relative position in the morphospace. A green polygon is used to represent the spread of species for each family.

**Figure 3:**
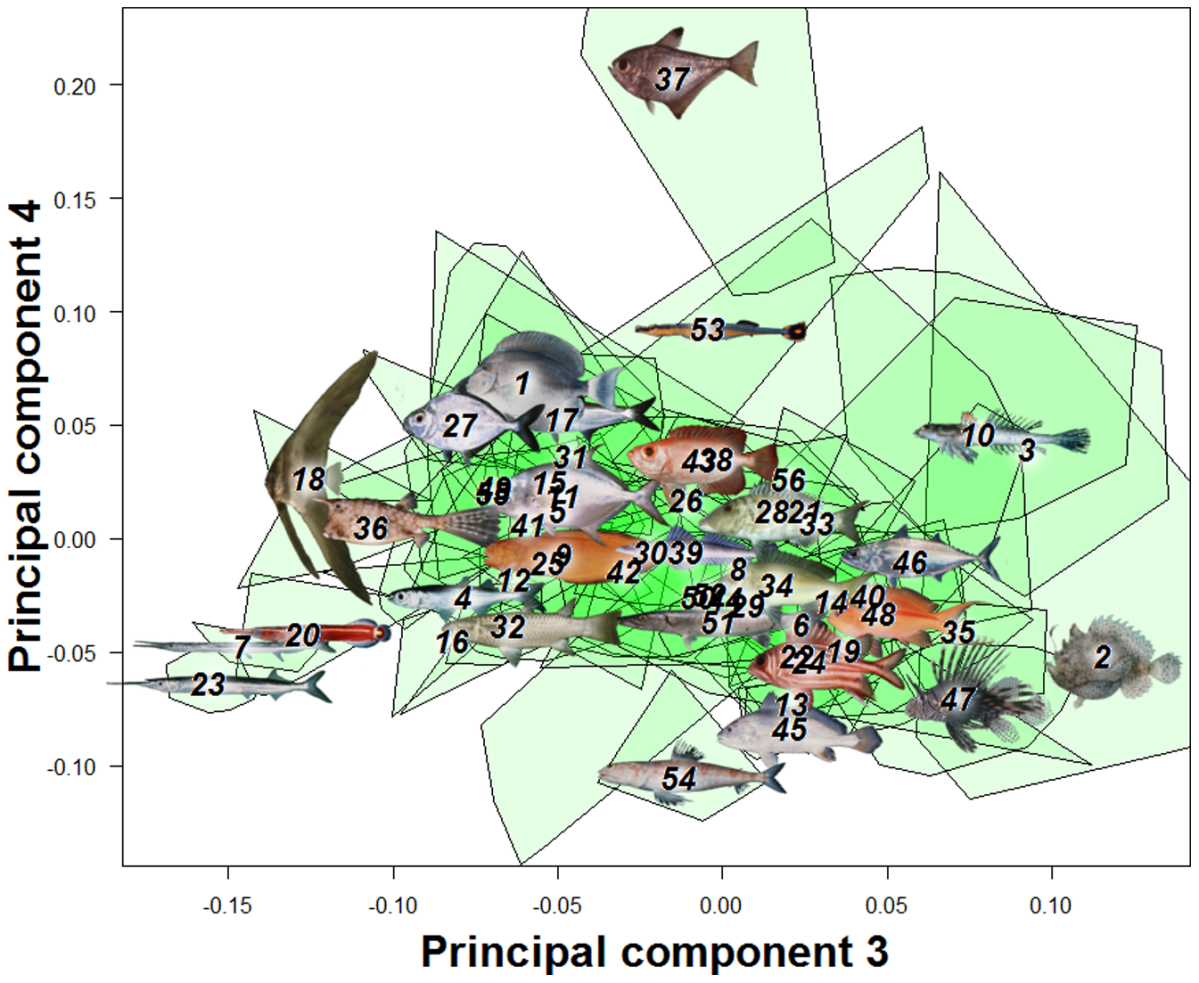
A principal component analysis (PCA) on overall fish shape data showed four main axes of shape deformation representing 13.23% and 6.87% for the third and fourth principal component of the overall shape variation in reef fishes. Fish pictures and family numbers are placed on the centroid of each family and used to identify their relative position in the morphospace. A green polygon is used to represent the spread of species for each family.

**Figure 4:**
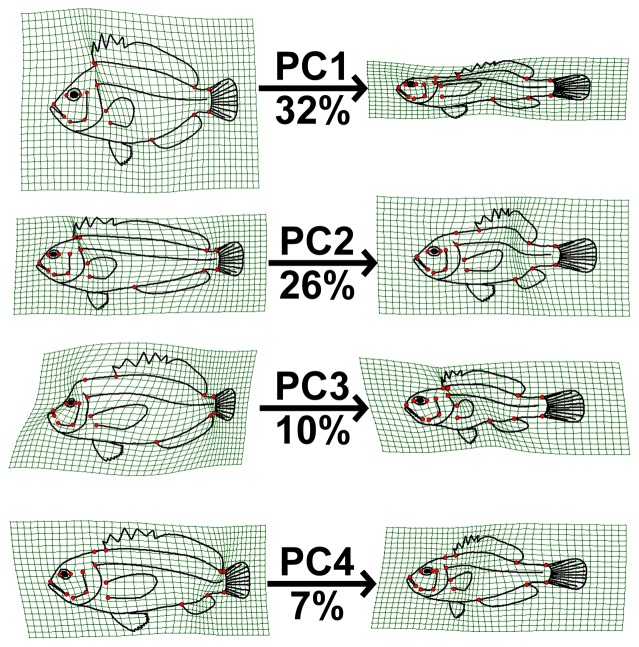
A grid line deformation along each of the four principal components represented in **fig. 2**&**3** is presented to describe deformation along these axes. Percentages represent variance explained by each axes.

The first three principal components from a principal components analysis (PCA) on landmark data were recovered as meaningful with a broken-stick analysis. For convenience of visual presentation (Fig. 2 & 3), PC4 is reported and discussed in the following. Together the first four PCs explained 75.3% of the shape variation in the overall dataset. The first axis (32.1% of total variance) represents a contrast between deep-bodied and elongate shapes (Fig. 2 & 4). For example, extremes on this axis are the extremely deep-bodied Ephippidae at the low end and the slender-bodied Belonidae at the high end. Within the head region, the change in body depth is mainly accomplished by compression or dilation of the dorsal region of the head, not by a global dorsoventral change. The position and shape of the mouth, preoperculum and eye barely change along PC1. There is also some posterior movement of the anterior edge of the dorsal fin as the body becomes elongate and slender. The second PC (26.2%) reflects a contrast between long and short dorsal and anal fins (Fig. 2 & 4, e.g. at the extremes are some Blenniidae with elongate fins vs some Tetraodontidae species with very short fins). The third axis (13.2%) reflects a contrast between small and large mouths, a short and pronounced facial region, and a rotation of the pectoral fin from vertical to a more horizontal orientation (Fig. 3 & 4). At extremes of this axis are small-mouthed Ephippidae and very large-mouthed Antennariidae species. Finally, fish with a high score on the fourth PC (6.87%) have a slight upward rotation of the mouth, an increase of the anal fin basal length, and a decrease of the dorsal fin basal length (Fig. 3 & 4). For example, Pempheridae have high PC4 scores while Synodontidae are at the other extreme.

**Figure 5:**
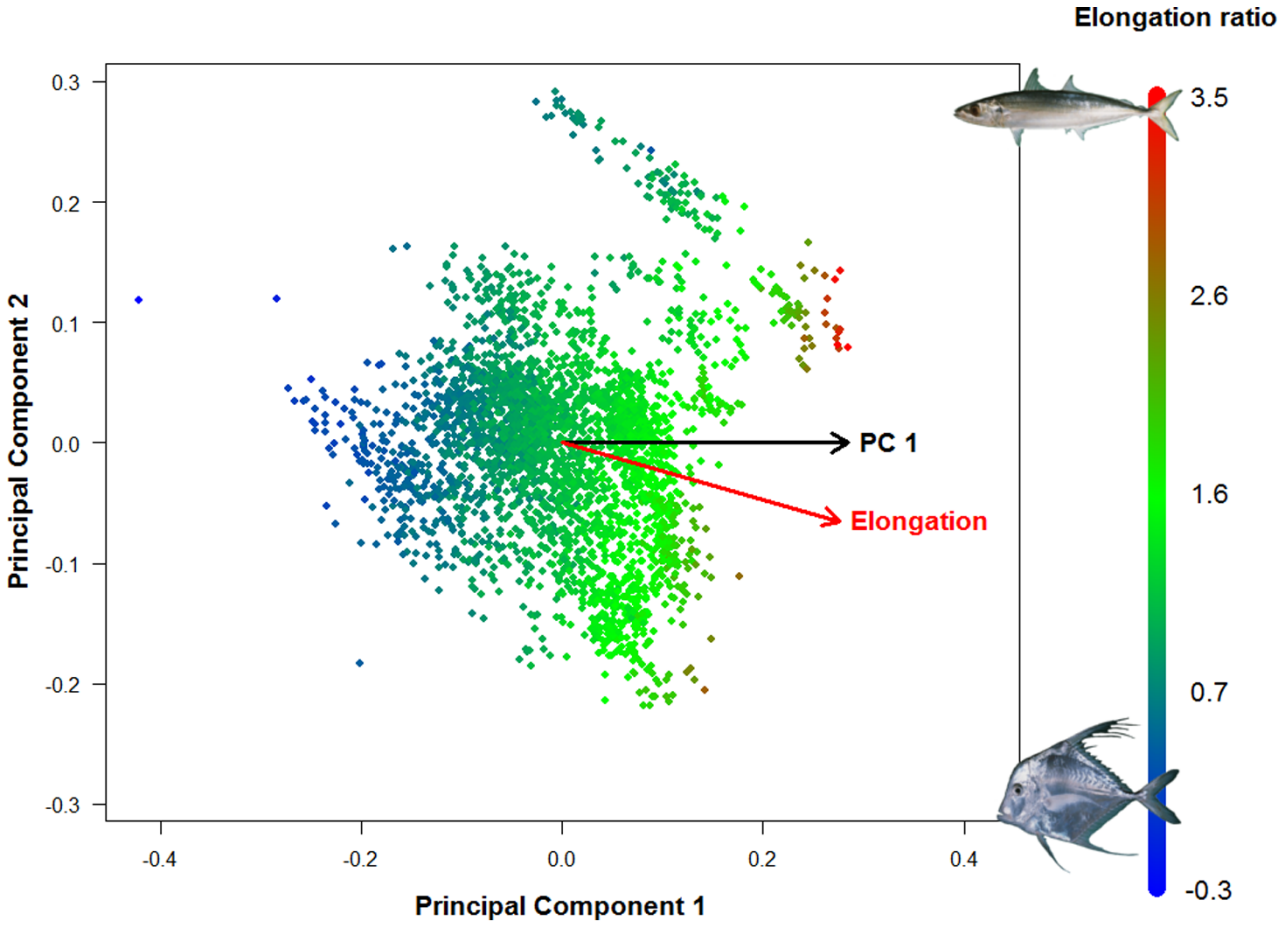
The fish morphospace presented in **figure 2A** is represented here with each species colored by their elongation ratio (**Fig. 1**). High ratios represent elongate fish while low ratio represents deep bodied fishes. The PC1 and elongation vectors are also represented on this morphospace. Based on our resampling procedure, the small angle between these two vectors (14.35°) indicate that elongation is significantly associated to the main axis of shape diversification (p<0.001).

**Figure 6:**
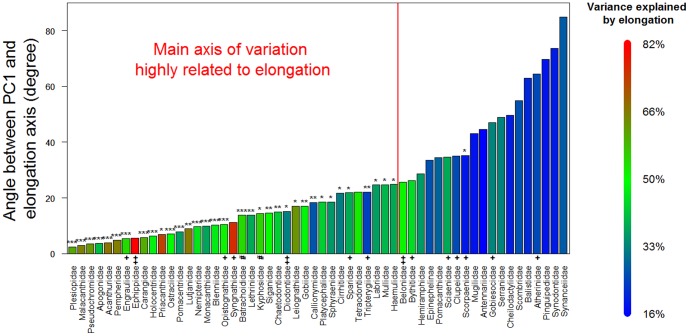
A majority of families (66%) have their main axis of shape variation strongly associated to elongation. The angles between PC1 and elongation vectors presented in figure 4 are presented here for each family on the y axis. The percentage of shape variation explained by elongation for each family is represented by the color of the bar (colors legend on the right). The significance of the association between PC1 and pure elongation is represented by the stars above bars (*p<0.05, **p<0.01, ***p<0.001). + represents the poorly sampled taxa (<20% of total accepted number of species), ++ represent taxa with less than 10 species sampled and # represent families poorly sampled and with less than 10 species.

The first PC of the overall data set differs from an axis of pure elongation (the axis defined by the ratio of body length/maximum body depth) in the morphospace by only 14.4 degrees, and these two vectors are significantly associated (p<0.001, Fig. 5). Looking at shape change within each family, in 66% of the families the main axis of shape diversification cannot be distinguished from elongation (Fig. 6).

### Patterns of shape change within families

**Figure 7:**
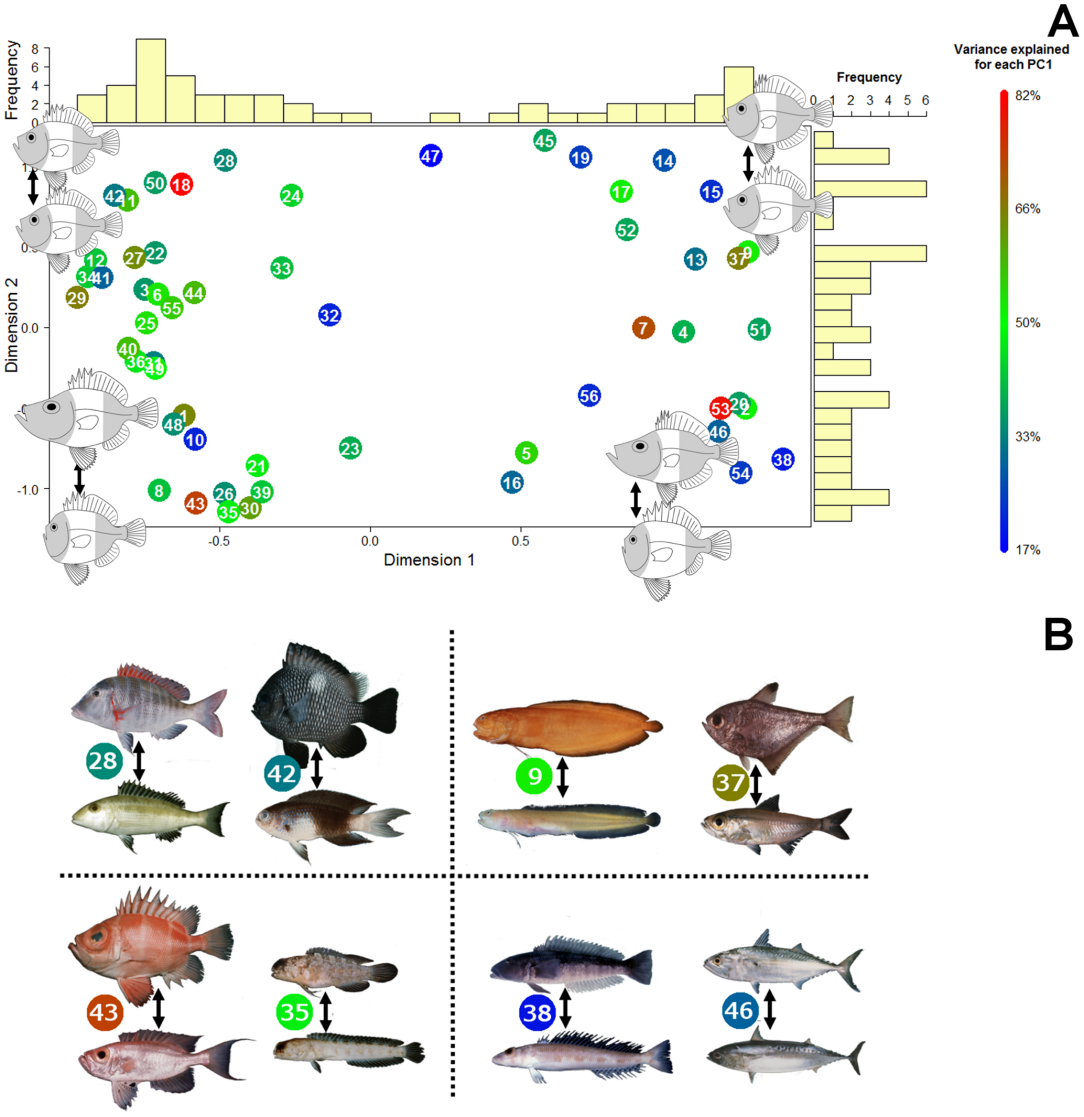
The two first axes of a principal coordinate analysis (PCoA) conducted on differences in orientation of the first PC for each of the 56 families with a representation of the deformation associated for each corners (A). The first principal component of shape was extracted for each family (Fig. 2). Then, the pairwise angles among the different families PC1 were used as a metric to quantify similarities in shape change among families. Finally a principal coordinate analysis was used to visualize similarity among families in the primary axis of body shape change. Families that fall close together in this analysis express similar patterns of shape change on their family-specific PC1. Colors represent the variance explained by PC1 for each family and the numbers inside each symbol corresponds to the family names listed in Table 1. Barplots reveal a bimodal distribution of families on the first axis. Examples of family changes along their first PC are represented for the four corners (B). It is important to note that the position of the poorly sampled families may change if sample size increase (This concern families 4, 6, 7, 9, 15, 16, 17, 18, 20, 25, 35, 47, 49, 50, 52 and 56).

The principal coordinates analysis (PCoA) on the PC1 vectors for each of the 56 families reveals major patterns of diversity in how shape changes within family. The first two axes from the PCoA account for 18.6% of the overall variation in shape change among families. The first axis (10.9%, Fig. 7A) represents, at the low end, families that diversify by similar change in all body regions, while on the high end of that axis, families show changes in the relative proportion of the body occupied by the precaudal and caudal regions. Families show a bimodal distribution along this axis, clustering toward the extremes (Fig. 7A). The second axis (7.8%, Fig. 7A) represents, on its low end, families that show changes in the proportion of the body made up by the head and postcranial regions. On the high end of this axis, families change body shape (i.e. mainly depth) without affecting the head and body proportions (Fig. 7A), while there is change in the proportion made by trunk and caudal regions. Examples of families in the four corners of this plot include Opistognathidae (family 35) and the Priacanthidae (family 43) in the bottom left corner, that show very little change in head shape, relative to the postcranial part of the body which experiences elongation while conserving trunk and caudal region proportions (Fig. 7B). In other words, these families undergo elongation in the postcranial region, but not the head. Lethrinidae (family 28) and Pomacentridae (family 42), in the upper left corner, show changes in body depth while maintaining constant proportions of the body made by the head, trunk and caudal regions. Pempheridae (family 37) and Bythitidae (family 9), in the upper right corner, undergo elongation of the caudal region and compression of the trunk as the body becomes deeper. Finally, Pinguipedidae (family 38) and Scombridae (family 46), in the lower right corner, show a compression of the trunk region as the body becomes more elongate (Fig. 7B). Care should be taken in interpreting these results because amplitude of the variation within each family may vary (amplitude is coded by color of dots in Fig 7B).

## Discussion

The dominant axis of shape variation in Indo-Pacific reef fishes contrasts deep-bodied fish at one extreme (e.g. *Platax*, *Chaetodon, Zebrasoma, Zanclus*) with slender, highly elongate forms at the other extreme (e.g. *Syngnathus*, *Platybelone, Hemirhamphus, Synodus*). This pattern was repeated within two-thirds of the families investigated indicating that the overall importance of elongation emerges from an important role at different phylogenetic scales. Nevertheless, we identified considerable diversity in the anatomical basis of elongation, suggesting underlying variation in the developmental basis of these changes and the functional consequences of body shape.

### Shape diversification in fishes

Reef fish morphological diversity is enormous, yet we identified two axes that account for more than half of the total variation in body shape: these axes capture variation in relative body depth and the length and position of the median fins. Considering the landmarks we chose in this study (eye position, mouth size, fin position, etc.), and the amount of diversity present in fish shape [41], this result was neither obvious nor inevitable. For example, given the diversity of fish feeding mechanisms [42]–[47], it would be plausible for the main axis of shape variation to be related to changes in head morphology, particularly because nine out of 17 landmarks were selected to measure shape of functionally important head features. In fact, head shape variation was not strongly expressed until the 3^rd^ and 4^th^ PCs. The third and fourth PCs (20.1% of the total shape variation combined) include head shape variation mainly expressed by mouth size and orientation. Overall, body elongation and fin position dominate shape diversity in our sample, while head deformation appears to be secondary in importance.

The dominance of the elongation axis could have emerged from a history in which changes in elongation took place before the modern lineages (families) were established. But this does not appear to be the case, as there is extensive overlap in the morphospace among most of the families (Fig. 2) and the pattern of variation in elongation dominates within families (Fig. 6). Another possibility is that elongation evolved multiple times in most of the extant clades. Variation at the broad phylogenetic scale of our entire sample could hide important axes of shape change specific to families, such as head deformations that would contribute to elongating or deepening body shape. We found elongation to be the dominant axis of variation at the scale of the overall sample, and for two-thirds of the families sampled (Fig. 6). However, this suggests that for about one-third of the families, evolutionary changes in shape are not strongly associated with body elongation.

The analysis of similarity among families in their primary axis of shape deformation (PC1) using a principal coordinates analysis [39] reveals diversity in how elongation is achieved. In this plot (Fig. 7), families cluster based on the nature of their primary axis of shape change, not based on their average shape. Note for example, Bythitidae and Pempheridae have nearly the same position in the PCoA plot, indicating similar patterns of shape change along PC1 for each family, even though the average shape of fish in the two families is quite different (Fig. 7B, top right).

The distribution of families in the principal coordinates space reveals that the main axes of fish shape variation are driven by different types of body deformation. The distribution of families in the space of the first two principal coordinate axes reveals one cluster of families in the upper left that elongate by proportional changes spread along the body. In the bottom left region of the plot are families that show proportional changes in the trunk and caudal regions but show changes in the head/post cranial proportions mainly with a bigger head as species become deep bodied. In the upper right region of the plot, families show proportional changes in the head/postcranial regions, but different changes to the proportions of trunk and caudal regions. Finally, in the bottom right, families elongate by a reduction in the contribution of the head and caudal region but stretch the trunk (Fig. 7). This diversity in how body regions are affected during elongation strongly suggests diversity in both the selective factors that cause these shape changes and the underlying developmental processes that effect the change.

The head, trunk, and caudal region were previously proposed to be three distinct developmental modules in fishes [5], [14]. Because we did not investigate within species variation, we will only discuss here cross-species variation and its implications for evolutionary modularity rather than developmental modularity [48], [49]. Ward and Brainerd [5] showed that the number of vertebrae in the trunk vs. the caudal region can vary independently across fish species, indicating some separation in the developmental regulation of the two body regions. A negative relationship between relative head length and body length has been shown to occur in moray eels [14], which appears to be different from the pattern in a number of other fish clades [5]. These results indicate the potential for head morphology to evolve independently from the postcranial region. Our results are consistent with the presence of three distinct body regions in fishes, and emphasize the diversity of changes that can happen in these three regions, particularly between the head and the postcranial regions (dimension 2, Fig. 7A). Furthermore, the variation in change among these three body regions in our sample of reef fishes is broadly consistent with patterns that have been found in a wide range of vertebrate groups [1], [50]–[53].

Changes in the relative length of the trunk and caudal regions is likely to frequently correspond to changes in the number of vertebrae found in each body region [5]. Families that have low scores on principal coordinate 1, such as Labridae, probably have a stable or proportional number of vertebrae in the trunk and caudal regions, while families with high scores on the first axis, such as Pempheridae and Bythidae, may show non-proportional changes in the number of vertebrae across species between the trunk and caudal region (Fig. 7A). Of course, this prediction relies on the assumption that the relative size of the trunk and caudal regions is based on differences in vertebrae number rather than a change in vertebrae length, an assumption previously confirmed in several fish groups [5], [14].

### Functional consequences of the elongation axis

The dominance of an axis of body elongation in our Indo-Pacific reef fish sample raises the question of why this axis of change is so important. What are the functional consequences of changing body elongation along this axis? Perhaps the most striking pattern is that ecological and habitat diversity found at each end of this shape axis is extremely high. There are no rigid associations between body elongation and habitat or trophic mode. For example, there are elongate taxa that are benthic (e.g. some members of Synodontidae, Gobiesocidae, Blenniidae, Gobiidae), others that are midwater (e.g. some Blenniidae, Syngnathidae) and still others are surface dwellers (e.g. Belonidae, Hemirhamphidae). It appears that depending on the circumstances, elongation may be an adaptation to a more benthic lifestyle (e.g. Callionymidae, Synodontidae) or it may be an adaptation to a more open-water lifestyle (e.g. the transition from lutjanids to caesionids). A number of studies of the consequences of more subtle changes in body shape have shown that changes to a more slender-bodied form accompany increases in steady swimming performance (reviewed in [54]). Thus, lineages employing axial undulation to propel themselves can be expected to become more elongate when they adopt a lifestyle of more active swimming in the water column.

At the other extreme on the axis of elongation are deep-bodied fish. A large number of these have small mouths and are specialized to feed by biting the benthos (e.g. Chaetodontidae, Acanthuridae, Pomacanthidae). Being deep-bodied with well-developed dorsal and anal spines, these fish gain considerable protection from gape-limited predators such as many serranids, carangids, and lutjanids. Although the association has not been thoroughly explored in the literature, our impression is that several species-rich groups of fishes in our sample show a trend within the family of more benthic-associated species being more deep-bodied (e.g. Sparidae, Haemulidae, Lutjanidae, Lethrinidae, Nemipteridae, Pomacentridae, Acanthuridae, Pomacanthidae). One traditional explanation for such a pattern is that deep-bodied fish may be at an advantage in unsteady locomotion, particularly with maneuverability [55], [56]. Both fast-start performance [54] and maneuverability are thought to be enhanced by a body form that provides increased lateral area for propulsion during fast starts, and where the fins are further away from the center of mass and thus provide greater mechanical advantage during turning maneuvers [57]. These advantages may be especially valuable to fish that propel themselves with lateral undulations of the axial column and are in close proximity to the physically complex reef. It is possible that evolutionary movement up and down the axis of body elongation is often associated with life-style changes that take advantage of the locomotion consequences of body shape.

Body elongation also has implications for feeding mechanisms. For taxa that depend on suction feeding, the deep-bodied shape typically reflects a greater cross-sectional area of epaxial musculature attaching to the back of the neurocranium [58]. This is an important determinant of the magnitude of suction pressure that a fish can generate when feeding [59], [60], indicating that evolutionary changes to a more deep-bodied shape often involve an increase in the capacity to generate suction pressure when feeding.

The nature of the internal basis of changes in body elongation has functional implications. As the body elongates this may be accompanied by proportional elongation of individual vertebrae or by increasing the number of vertebrae in the axial column [5], [13]. Elongate fish with many vertebrae tend to be highly flexible, while species with fewer and elongate vertebrae tend to be stiffer in lateral bending [61], [62]. One common feature of highly elongate forms with many vertebrae is a fossorial lifestyle, living among rocks or within sand or mud. This lifestyle is characteristic of moray eels and some temperate shoreline gobies, neither of which were sampled in our study. While we did not make internal measurements of skeletal elements in our study our unpublished observations suggest that members of Belonidae, Hemirhamphidae, and within Gobiidae, *Ptereleotris* are all elongate taxa with elongate vertebrae. These taxa have relatively stiff bodies and we suggest they maintain the capacity for high performance swimming by lateral undulation.

## Conclusions

Although reef fishes are tremendously diverse in body shape, 32% of the variation in form can be attributed to changes in elongation. The dominance of elongation emerges from a pattern that is seen both across the full range of our sample of 2939 species and within two-thirds of the families we studied. Shape variation along this axis has known consequences for locomotion and feeding performance, but associations with habitat use and trophic patterns are characterized by a very loose fit between body shape and niche. In other words, for a given body elongation, reef fishes exhibit astonishing ecological diversity. This apparent many-to-one mapping of body elongation to ecological niche suggests that extraordinary shape diversity of teleosts might not only be a consequence of the great age of their lineage, but could result from a combination of flexibility in developmental processes and the presence of many adaptive solutions to the challenges presented in their environment.

## Supporting Information

File S1 **R functions to calculate angles between PC1 and the axis of maximum elongation based on elongation ratio of each species; it could be any other continuous variable associated with each species, such as swimming speed or geographical gradient.** The function combines PCA and RDA. The code includes a function to calculate the angle and another function to test whether PC1 is significantly associated to the axis of maximum elongation using permutation.
(TXT)
